# SAFEGUARDING IN THE INTERNET OF THINGS AGE: A COMPREHENSIVE REVIEW OF SECURITY AND PRIVACY RISKS FOR OLDER ADULTS

**DOI:** 10.1093/geroni/igad104.2642

**Published:** 2023-12-21

**Authors:** Suleiman Saka, Sanchari Das

**Affiliations:** University of Denver, Denver, Colorado, United States; University of Denver, Denver, Colorado, United States

## Abstract

The advent of the Internet of Things (IoT) has brought about a technological revolution that has transformed the way we live and interact with the world. IoT technology has the potential to enhance the quality of life for individuals, particularly older adults, by enabling them to access and control devices remotely. However, IoT technology also raises significant security and privacy concerns, especially for older adults, who are often less technologically savvy and more susceptible to cyber threats. This paper provides a systematic review of the literature related to IoT technology for older adults and identifies key gaps in the security and privacy of these technologies. Our review analyzed a total of 116 papers related to IoT for older adults and focused on 25 that met our criteria. We found that most of the research in this area focuses on the health and medical sector, where IoT technology can be used to monitor the health and well-being of older adults. However, our analysis revealed that there are significant gaps in the security and privacy of IoT technology for older adults. To address these gaps, we provide actionable recommendations for enhancing the security and privacy of IoT technology designed for older adults. Our recommendations include the development of user-friendly interfaces, the use of secure and reliable communication protocols, the incorporation of data encryption and anonymization, and the establishment of regulatory frameworks to ensure the protection of personal data.

